# Association between mass media exposure and endorsement of HIV-infected female teachers' teaching: insight from 2014 Ghana Demographic and Health Survey

**DOI:** 10.1186/s12905-022-01705-1

**Published:** 2022-04-15

**Authors:** Francis Appiah, Justice Ofosu Darko Fenteng, Andrews Ohene Darteh, Felix Dare, Joel Afram Saah, Matthew Takyi, Patience Ansomah Ayerakwah, Kingsley Boakye, Edward Kwabena Ameyaw

**Affiliations:** 1 Berekum College of Education, Berekum, Bono Region Ghana; 2 Department of Population and Health, College of Humanities and Legal Studies, University of Cape Coast, Cape Coast, Ghana; 3 School of Public Health, Kwame Nkrumah University of Science and Technology, Kumasi, Ghana; 4 Department of Public Health, School of Allied Health Sciences, University for Development Studies, Tamale, Ghana; 5 Department of Optometry, University of Cape Coast, Cape Coast, Ghana; 6 Department of Epidemiology and Biostatistics, School of Public Health, Kwame Nkrumah University of Science and Technology, Kumasi, Ghana; 7 School of Public Health, Faculty of Health, University of Technology Sydney, Ultimo, Australia

**Keywords:** HIV/AIDS, Mass media, Ghana AIDS Commission, Public health, HIV infected female teacher

## Abstract

**Introduction:**

Ghana recorded 19,931 new annual HIV infections in 2018 translating to 14,181 AIDS-related deaths. Mass media is capable of ensuring the sustainability of initiatives aimed at curbing HIV/AIDS epidemic by reducing HIV/AIDS stigma and discrimination. The study aimed at exploring if mass media plays a role in whether HIV-infected female teachers should be allowed to teach in Ghana.

**Materials and methods:**

The study used women’s file of the 2014 Ghana Demographic and Health Survey (GDHS). The current study was restricted to 6025 women who had complete information on the variables analysed. Binary Logistic regression was conducted between mass media and the dependent variable whilst controlling for the effect of the significant covariates. The results were presented in odds ratio (OR) and adjusted odds ratio (AOR) at 95% confidence interval (CI). All analyses were carried out using STATA version 14.0.

**Results:**

Generally, 51% of the women mentioned that HIV-infected female teachers should be allowed to teach in Ghana. Women who accessed mass media had higher odds of endorsing that HIV-infected female teachersshould be allowed to teach compared to those without access to mass media [AOR = 1.37, CI 1.200–1.555], just as among those  with secondary or higher education compared to those with no formal education [AOR = 1.30, CI 1.102–1.529]. Rural inhabitants had decreased odds of proclaiming that HIV-infected female teachers should be allowed to continue teaching compared with urban residents [AOR = 0.83, CI 0.717–0.957].

**Conclusions:**

Endorsement of HIV-positive female teachers’ continued teaching was associated with access to mass media. This is suggestive that various mass media platforms could help to reach the public with HIV/AIDS-related information, particularly those that touch on stigma and discrimination, which will potentially increase women’s knowledge and shape their perceptions about persons living with HIV.

**Supplementary Information:**

The online version contains supplementary material available at 10.1186/s12905-022-01705-1

## Introduction

Human Immunodeficiency Virus/Acquired Immunodeficiency Syndrome (HIV/AIDS) is still a public health challenge, despite notable progress in information, diagnostics, treatment, and prevention of the disease in the last 2 decades [1]. Globally, about 32.7 to 44 million persons were known that have had HIV/AIDS in 2018 [2, 3] and further records indicate that over two in every three persons (69%) living with HIV/AIDS episodes are from sub-Saharan Africa (SSA) [3, 4]. With persons living with HIV/AIDS in SSA stalling between 17.5 and 21.1 million [4], this could threaten the prospects of SSA in realising Sustainable Development Goal 3 (SDG-3) [5]. SSA alone accounted for about 74% of the 1.5 million worldwide AIDS-related fatalities recorded in 2013 [6] and the picture of new HIV/AIDS infections is almost the same. Ghana’s experience of HIV/AIDS is almost the same as SSA. According to the Ghana AIDS Commission, Ghana has seen a modest decline in HIV infection from 1.77% to 1.67% among the adult population between 2015 and 2019 [7]. However, further estimates suggest that Ghana recorded 19,931 new annual HIV infections in 2018 whereas, in the same year, about 14,181 AIDS-related deaths were recorded [7].

To respond to HIV/AIDS, the Government of Ghana implemented institutional structures across national and local level healthcare systems and deduced strategies targeted at controlling HIV/AIDS [8, 9]. With the Ministry of Health serving as national technical support, a key tool employed was Information, Education and Communication (IE&C) campaigns around 1994 using television and radio spot advertisements and print media [8, 9]. After this media intervention, efforts have been made to augment IE&C initiatives [8, 9]. The nation is striving towards achieving the United Nations Programme on HIV/AIDS’ 90–90-90 targets which emphasise that, 90% of persons living with HIV should know their status, 90% of those diagnosed should be on treatment, and 90% of those on treatment should be virally suppressed within 12 months of initiating treatment [10]. In September 2016, the government of Ghana adopted the World Health Organization policy of “treat all”—which is the provision of antiretroviral treatment to all persons living with HIV (PLHIV), irrespective of their CD4 counts [11, 12].

The role of mass media concerning HIV/AIDS epidemic is critical as the former can be used for attitudinal and behavioural changes associated with HIV/AIDS [13, 14]. For instance, television campaigns usually yield a strong impact in terms of HIV/AIDS awareness, transmission knowledge, interpersonal communication, and behavioural change, as opposed to campaigns using other channels, such as radio or print media [15]. Additionally, one of the desired effects of mass media interventions is an increase in HIV/AIDS knowledge [16].

Strategies targeted at HIV/AIDS control involving the media are worth implementing since a critical area affecting the campaign against HIV/AIDS is lack of knowledge, misconceptions about HIV transmission, and low level of education which lead to fear and negative attitudes toward PLHIV [17]. Various factors contribute to these misconceptions, stigmatization, and discrimination against PLHIV including lack of understanding of the disease, misconceptions about transmission, and inaccessibility of treatment [13].

As a result, combating stigma and discrimination directed toward PLHIV, such as a female teacher who is infected with HIV, is one of the five imperatives that ensure the sustainability of initiatives aimed at curbing HIV/AIDS epidemic [18]. Notwithstanding, a critical area that is less explored is the association between mass media on HIV/AIDS-related stigma in Ghana. Prior studies in Ghana have looked at mass media and HIV/AIDS prevention [9], adolescents’ exposure to mass media campaign messages on HIV/AIDS [19], the HIV epidemic, and the United States President’s Emergency Plan for Emergency Relief’s (PEPFAR) response to the epidemic [12] and a gendered analysis of living with HIV/AIDS in the Eastern Region [20]. Additionally, a multi-country study that assessed the effects of exposure to mass media on HIV-related stigma also used the fifth round of DHS dataset usually captured around 2006–2011 which presents a challenge to understanding current knowledge about the effect of mass media on HIV stigma [21].

HIV stigma negatively impacts the supply of teachers and retention rate of students [22] and HIV-related stigma could cause PLHIV to lose social value and recognition [23]. Prior research suggests that social stigma affects emotions, cognitions, and behaviour of PLHIV [24] whereas interpersonal processes cause PLHIV to internalize and anticipate stigma, which subsequently results in negative individual-level health consequences, ranging from depression to poor medication and visit adherence [23]. Therefore, this study is motivated to explore the association between mass media and whether HIV positive female teachers be allowed to teach in Ghana using a current and nationally representative survey. The study hypothesised that women who are exposed to mass media will have lower odds to affirm that HIV positive female teachers be allowed to teach in Ghana. The findings of the study could constitute a basis for planning activities aimed at the prevention and control of HIV/AIDS and addressing stigmatisation and discrimination directed at PLHIV [25].

## Materials and methods

### Data source

The study used women’s file of the 2014 Ghana Demographic and Health Survey (GDHS),  since it is the current version of all GDHS as part of the global Demographic and Health Survey (DHS) Program. The GDHS made use of three questionnaires based on standard DHS questionnaires namely the Household Questionnaire, the Woman’s Questionnaire, and the Man’s Questionnaire. Issues covered in the woman’s questionnaire included HIV/AIDS and other sexually transmitted infections (STIs), delivery, postnatal care, and other essential population health indicators. The survey was implemented by the Ghana Statistical Service (GSS), the Ghana Health Service (GHS), and the National Public Health Reference Laboratory (NPHRL) of the GHS whereas ICF International provided technical assistance through The DHS Program. The survey employed a stratified probabilistic sampling method thereby ensuring an equal chance of participation for eligible women in Ghana. Overall sampling procedures are detailed in the 2014 GDHS report [26]. The 2014 GDHS identified 9656 eligible women (15–49 years) and successfully interviewed 9396 eligible women, at a response rate of 97.3%. Meanwhile, the current study was restricted to 6025 women who had complete information for analysis.

### Derivation of dependent variable

The main dependent variable for the study was whether an HIV infected female teacher should be allowed to continue teaching. In the survey, respondents were asked, “In your opinion, if a female teacher is HIV positive but not sick, should she be allowed to continue teaching in the school? The responses were: ‘should be allowed’, ‘should not be allowed’, and ‘don’t know/not sure/depends.’ We excluded all persons who indicated ‘don’t know/not sure/depends’ due to their uncertainty about whether an HIV infected female teacher should be allowed to teach or otherwise. Consequently, we recoded ‘should be allowed’ and ‘should not be allowed’ into ‘1’ and ‘0’ respectively.

### Derivation of independent variable

The principal independent variable was mass media. This was derived from three cardinal variables: frequency of reading newspaper/magazine; frequency of listening to the radio; and frequency of watching television which was asked during the 2014 GDHS. Each of these variables had three responses: ‘not at all’, ‘less than once a week’, and ‘at least once a week’. A composite variable was created whereby all ‘less than once a week’ and ‘at least once a week’ responses were categorised as having access to mass media whilst ‘not at all’ was considered as not having access to mass media.

### Derivation of covariates

Ten co-variates of theoretical significance to the study were added to our analysis: age; educational attainment; residence; marital status; religion; wealth status; occupation; ethnicity; parity; and partner’s educational attainment. To enhance the readability of the results, educational attainment was recoded into ‘no education = 1’, ‘primary = 2’, ‘secondary or above = 3’. Marital status was recoded into ‘never married = 1’ ‘married = 2’ ‘cohabiting = 3’, ‘widowed = 4’ ‘divorced = 5’ and ‘separated = 6’ whilst religion of affiliation was recoded into ‘Christianity = 1’ ‘Islam = 2’ ‘Traditional = 3’ and ‘no religion = 4’. For precision in responses, wealth status was recoded into ‘poor = 1’ ‘middle = 2’ and ‘rich = 3’ whereas occupation was recoded into ‘not working = 1’ and ‘working = 2’. Parity was recoded into ‘zero birth = 1’, ‘one birth = 2’, ‘two births = 3’, ‘three births = 4’, and ‘four or more births = 5’. Finally, the partner’s educational attainment was recoded into ‘no education = 1’, ‘primary = 2’, ‘secondary or above = 3’, and ‘don’t know = 4’.

### Analytical procedure

The following procedures were used in analysing the data. To begin with, the percentage of women who indicated that female teachers infected with HIV should be allowed to continue teaching or otherwise was computed. This was followed by a cross-tabulation between mass media, socio-demographic characteristics, and endorsement of HIV infected female teachers teaching. A chi-square test of independence was carried out to ascertain socio-demographic variables that have a significant association with the outcome variable (Table 1). Significant variables were included in the inferential analysis. Binary Logistic regression was carried out between mass media and the dependent variable (Model I). The effect of significant covariates was controlled in Model II (Table 2). The results were presented in odds ratio (OR) and adjusted odds ratio (AOR) at a 95% alpha threshold with a two-tailed confidence interval (CI). Variance inflation factor (VIF) was applied to test for multi-collinearity between the explanatory variables and the results indicated that the independent variables were not highly correlated (Mean VIF = 1.45, Maximum VIF = 2.44, Minimum VIF = 1.02) (Additional file 1: Appendix S1). Also, the weighting factor (v005/100,000) inherent in the dataset was applied to cater for over and under sampling errors. Finally, the ‘linktest’ command was used to assess the fitness of the model and the results indicated that the model was well specified (Table 2). All analyses were carried out with the STATA version 14.0.

**Table 1:** Socio-demographic characteristics and HIV/AIDS-related stigma (N = 6025). *Source*: GDHS 2014

Variables	Weighted (N)	Weighted (%)	HIV infected female teachers should be allowed to continue teaching		
			Not allowed (%)	Allowed (%)	X^2^ (*p*-value)
*Access to mass media*					191.7075 (0.000)
No	1901	32	60	40	
Yes	4124	68	41	59	
*Age*					12.271 (0.056)
15–19	111	2	58	42	
20–24	657	11	50	50	
25–29	1116	19	45	55	
30–34	1186	20	48	52	
35–39	1162	19	47	53	
40–44	981	16	49	51	
45–49	812	13	50	50	
*Education attainment*					190.591 (0.000)
No education	1513	25	57	43	
Primary	1169	19	55	45	
Secondary +	3342	56	39	61	
*Residence*					197.665 (0.000)
Urban	3104	52	38	62	
Rural	2920	48	57	43	
*Marital status*					37.921 (0.000)
Married	3793	63	47	53	
Cohabiting	1295	22	55	45	
Widowed	242	4	44	56	
Divorced	269	4	38	62	
Separated	426	7	45	55	
*Religion*					88.551 (0.000)
Christianity	4769	79	47	53	
Islam	931	16	47	53	
Traditional	135	2	76	24	
No Religion	190	3	67	33	
*Wealth status*					294.907 (0.000)
Poor	2103	35	59	41	
Middle	1251	21	47	53	
Rich	2671	44	34	66	
*Occupation*					0.254 (0.614)
Not Working	734	12	47	53	
Working	5291	88	48	52	
Ethnicity					222.614 (0.000)
Akan	3031	50	45	55	
Ga/Dangme	471	8	50	50	
Ewe	788	13	54	46	
Guan	146	2	47	53	
Mole-Dagbani	924	15	45	55	
Grusi	173	3	33	67	
Gurma	327	6	81	19	
Mande	55	1	56	44	
Other	110	2	49	51	
*Parity*					104.662 (0.000)
Zero birth	366	6	33	67	
One birth	916	15	41	59	
Two births	1164	19	44	56	
Three births	1048	18	46	54	
Four or more births	2531	42	55	45	
*Partners’ education attainment*					155.061 (0.000)
No education	1101	18	59	41	
Primary	526	9	57	43	
Secondary +	4258	71	42	58	
Don’t Know	140	2	56	44

**Table 2:** Binary logistic regression results on mass media and whether HIV infected female teacher should be allowed to teach or otherwise. *Sources*: GDHS 2014.

Variable	Model I		Model II	
	OR	95% CI	AOR	95% CI
*Mass media*				
No access	Ref	1.1	Ref	1.1
Access	2.10***	[1.892–2.339]	1.37***	[1.200–1.555]
*Education attainment*				
No education			Ref	1.1
Primary			0.92	[0.779–1.089]
Secondary +			1.30**	[1.102–1.529]
*Residence*				
Urban			Ref	1.1
Rural			0.83**	[0.717–0.957]
*Marital status*				
Married			Ref	1.1
Cohabiting			0.72***	[0.622–0.837]
Widowed			1.33*	[1.015–1.744]
Divorced			1.42*	[1.073–1.876]
Separated			0.98	[0.774–1.241]
*Religion*				
Christianity			Ref	1.1
Islam			0.84*	[0.706–0.993]
Traditional			0.62*	[0.420–0.905]
No Religion			0.72*	[0.530–0.988]
*Wealth status*				
Poor			Ref	1.1
Middle			1.23*	[1.040–1.442]
Rich			1.61***	[1.327–1.949]
*Ethnicity*				
Akan			Ref	1.1
Ga/Dangme			0.80	[0.627–1.019]
Ewe			0.80*	[0.672–0.962]
Guan			1.14	[0.825–1.582]
Mole-Dagbani			1.87***	[1.562–2.239]
Grusi			2.67***	[1.996–3.565]
Gurma			0.44***	[0.328–0.590]
Mande			1.13	[0.672–1.905]
Other			1.12	[0.720–1.736]
*Parity*				
Zero birth			Ref	1.1
One birth			0.75*	[0.564–0.989]
Two births			0.64**	[0.488–0.844]
Three births			0.64**	[0.483–0.843]
Four or more births			0.57***	[0.439–0.743]
*Partners’ education attainment*				
No education			Ref	1.1
Primary			1.10	[0.874–1.326]
Secondary +			1.38***	[1.161–1.631]
Don’t Know			0.92	[0.614–1.380]
*Model specification*				
Number of observations			6025	
LR *X*^*2*^			687.98	
*X*^*2*^			0.000	
Pseudo *R*^*2*^			0.082	
Log likelihood			− 3828.241	
_hat			0.99*** [0.916–1.081]	
_hatsq			− 0.13 [− 0.095 to 0.069]	

OR, Odds Ratio; AOR, Adjusted Odds Ratio; CI, Confidence Interval in square brackets; Ref, Reference Category

*p < 0.05; **p < 0.01; ***p < 0.001

### Ethical considerations

The study depended on an already existing dataset and therefore we were not directly involved in the data collection activities. Meanwhile, we requested the dataset from the DHS Programme’s website and after assessing our intent to use the dataset, they granted us approval to download and use the dataset. However, ethical considerations applicable to the survey can be found in the 2014 GDHS full report [26]. The dataset upon which the current study drew its findings and conclusions is publicly available at www.measuredhs.org.

## Results

### Descriptive results for the study

Generally, a little above half of the women (3102: 51%) mentioned that female teachers infected with HIV should be allowed to teach in a school, with a substantial proportion (2923: 49%) of them claiming otherwise (data not shown).

Table 1 depicts results on mass media, socio-demographic characteristics, and whether an HIV-infected female teacher should be allowed to continue teaching in a school or otherwise. Fifty-nine percent of those with access to mass media mentioned that female teachers infected with HIV should be allowed to continue teaching in a school. It was noticed that those who mention that female teachers infected with HIV should be allowed to continue teaching in a school peaked among those aged 25–29 (55%), those with secondary or higher education (61%), urban residence (62%) as well as the divorced (62%).

Christian (53%) and Islamic (53%) women were the highest to mention that female teachers infected with HIV should be allowed to continue teaching. The analysis also indicated that the rich (66%), women not working (53%), Grusi (67%), and those at parity zero (67%) were the highest to mention that female teachers infected with HIV should be allowed to continue teaching in a school. Additionally, those whose partners had attained secondary or higher education (58%) were the highest to state that female teachers infected with HIV should be allowed to continue teaching in a school. Finally, the results of the chi-square test indicated that, except for age and occupation, all the independent variables had a statistically significant association with the outcome variable (Table 1).

### Inferential results of the study

Table 2 displays the inferential results of the study. It was found that those who had access to mass media had higher odds of endorsing that female teachers infected with HIV should be allowed to continue teaching as compared to those without access to mass media [OR = 2.10, CI 1.892–2.339] and persisted after controlling for confounding variables [AOR = 1.37, CI 1.200–1.555]. It was also noted that those who had completed secondary or higher education had a higher likelihood to affirm that female teachers infected with HIV should be allowed to continue teaching compared to those with no formal education [AOR = 1.30, CI 1.102–1.529]. Also, those in rural areas had lesser odds to proclaim that female teachers infected with HIV should be allowed to continue teaching in a school compared with urban residents [AOR = 0.83, CI 0.717–0.957]. Again, comparatively, those cohabiting had lesser odds to endorse that female teachers infected with HIV should be allowed to continue teaching in a school compared with the married [AOR = 0.72, CI 0.622–0.837].

The analysis revealed that traditionalists were least probable to support that female teachers infected with HIV should be allowed to continue teaching in a school as compared with Christians [AOR = 0.62, CI 0.420–0.905]. It was evident that the rich had a higher tendency to endorse that female teachers infected with HIV should be allowed to continue teaching in a school as compared with the poor [AOR = 1.61, CI 1.327–1.949], just as among the Grusi as compared to the Akan [AOR = 2.67, CI 1.996–3.565]. Additionally, those at parity four or more were least inclined to endorse that female teachers infected with HIV should be allowed to continue teaching in a school as compared with those at parity zero [AOR = 0.57, CI 0.439–0.743]. Also, those whose partners had completed secondary or higher education were most probable to support that female teachers infected with HIV should be allowed to continue teaching in a school compared with those whose partners had no formal education [AOR = 1.38, CI 1.161–1.631]. Finally, it was found that the model was well specified (Table 2).

## Discussion

Studies on HIV-related stigma/discrimination have established that stigma tends to be higher among individuals with low media exposure than among those with high media exposure [21]. In light of this, the present study investigated the association between mass media exposure and whether HIV-infected female teachers should be allowed to teach or otherwise in Ghana. The principal finding of the study was that those who had access to mass media were more probable to endorse that female teachers infected with HIV should be allowed to continue teaching in school compared to those without access to mass media. Our findings coincide with various studies conducted in other parts of sub-Saharan Africa and Asia where it was revealed that media use was generally associated with low levels of HIV-related stigma and lessened the gap between individuals with high and low educational levels [21, 27, 28]. Similarly, in the Greater Accra, Ashanti, and Upper East regions, it was suggested that mass media channels can effectively address HIV-related stigma on a national scale [29]. Arguably, adequate knowledge and awareness of disease are the key prerequisites for its prevention and control, given that adequate knowledge is a basis for adopting the appropriate attitudes and practices [30–32]. This lays credence to the fact that employing television, radio, newspapers, and other forms of mass media to carry out HIV-related stigma/discrimination-related socio-behavioural education is needful. At least, exposing communities to HIV-related stigma-related information could significantly enhance community knowledge and understanding of the disease.

Another interesting finding was that those who had completed secondary or higher education had higher odds to affirm that female teachers infected with HIV should be allowed to continue teaching in a school as opposed to those with no formal education. The positive effects of formal education on HIV knowledge have been supported by many other studies. For instance, in Nigeria and Kenya, it was known that those with higher levels of education and with a higher wealth index are more sympathetic toward people living with HIV (PLHIV) [33, 34]. The association between formal education and knowledge of health-protective behaviour is often explained by the fact that education increases cognitive abilities, numeracy, and decision-making abilities and therefore improves abilities to engage in health-protective behaviours [35]. Specifically, formal education influence HIV and AIDS knowledge by providing people with the requisite information to protect themselves from infection.

It was found that those in rural areas were less likely to proclaim that female teachers infected with HIV be allowed to continue teaching in a school compared with urban residents. Previously conducted studies have consistently indicated that mass media was found to be stronger among urbanites rather than among rural residents which could lead to a widening gap between the two groups in the endorsement of HIV-related stigma [21, 33, 36]. The results imply that the social location in which this attitude prevails might be visualized at the intersection of other multiple identities. Similarly, the fact that stigma is high among people from rural communities is an indication that PLHIV in such communities could experience high levels of stigma. The foregoing study also revealed that, comparatively, those cohabiting had lesser odds to endorse that female teachers infected with HIV should be allowed to continue teaching in a school compared with the married. This could be attributable to pre-marital preparation and counselling organized for couples before marriage. Before marriage is contracted, couples go through HIV/AIDS voluntary testing and counselling which raise their understanding of HIV/AIDS.

In agreement with previous studies [37, 38], the current study revealed that traditionalists were least probable to support that female teachers infected with HIV should be allowed to continue teaching in a school as compared to Christian. A Ghanaian-based study also indicated that women who identified themselves as Christians were more knowledgeable about modes of HIV transmission than women who followed African traditional religion or were not religiously affiliated [37]. This is suggestive that a probable relationship exists between religious affiliation and levels of knowledge about HIV/AIDS. This finding is in line with a study conducted in Tanzania, where religious beliefs played a major role in shaping people's perspectives on HIV and PLHIV [38]. Nevertheless, a study in Mozambique found protestant women to have more comprehensive HIV and AIDS knowledge than their Catholic counterparts [39]. Such a finding suggests that policymakers need to pay more attention to the non-Christian groups in their HIV/AIDS campaigns as well as consider the differences existing among Christian groups.

The rich had a higher propensity to endorse that female teachers infected with HIV should be allowed to continue teaching in a school as compared to the poor. The result is in line with a study in Nigeria and Kenya which found that those in the higher wealth index are more sympathetic toward PLHIV [33, 34]. Probably, those with higher wealth status might own and have access to varied mass media platforms, hence, exposing them to various HIV/AIDS information. Our finding is similar to a previous study in Bangladesh in which the authors found that rich women had a higher likelihood of exposure to media and HIV/AIDS information [36]. Similarly, those whose partners had completed secondary or higher education were most probable to support that female teachers infected with HIV should be allowed to continue teaching in a school compared with those whose partners had no education. This could be due to cascading effect of partner’s knowledge on the couple as it is known that there is higher awareness among the educated and wealthy ones on the prognosis of PLHIV and the availability of antiretroviral treatments [34].

Finally, those at parity four or more were least inclined to endorse that female teachers infected with HIV should be allowed to continue teaching in a school as compared with those at parity zero just as among the Grusi as compared to the Akan. This could be due to diverse beliefs, perceptions, and practices held by various ethnic groups. However, evidence suggests that eliminating stigma and discrimination will result in higher acceptance of PLHIV by community members [40]. Therefore, promoting positive and acceptable attitudes toward PLHIV among diverse ethnic groupings and mothers of varying parity in Ghana is needful. Admittedly, the current study failed to provide probable reasons for the observed variations. Therefore, a study, preferably, qualitative study is needed to understand the phenomenon.

### Strengths and weaknesses of the study

This study investigated the association between mass media and HIV-related stigma in Ghana and it is the first of its kind to have done that. Also, the study depended on survey data from the former principal ten administrative regions in Ghana, hence representing the views of women in Ghana. The probability sampling approach employed in the survey aided to reduce sampling biases. The study further employed rigorous analytical procedures thereby enhancing the robustness of the results. However, a causal relationship cannot be established due to the cross-sectional nature of the survey. Also, issues surrounding HIV/AIDS, especially stigma and discrimination are delicate, respondents might be biased in their responses due to social desirability bias. The study also focused on females only and the findings and conclusions are based on views of females excluding males.

## Conclusions

Endorsement of HIV infected female teachers’ continued teaching was associated with access to mass media. This is suggestive that various mass media platforms could help to reach women with HIV/AIDS-related information, particularly information that touch on stigma and discrimination. Witnessing that HIV/AIDS is more prevalent among rural folks is an indication that, the Ghana AIDS Commission and other policymakers should intensify HIV/AIDS-related stigma programmes among rural dwellers to shape their perception and behaviours towards HIV/AIDS. Similar socio-behavioural sensitization programs need to focuse on the less educated and those found within poor socioeconomic category to address this problem.

## Supplementary Information

**Additional file 1.** Multi-collinearity test results.

## Data Availability

The datasets generated and/or analysed for this study are available in the Measure DHS repository at www.measuredhs.org.

## Abbreviations

AIDS: Acquired Immuno-Deficiency Syndrome
AOR: Adjusted Odds Ratio
GDHS: Ghana Demographic and Health Survey
GSS: Ghana Statistical Service
GHS: Ghana Health Service
HIV: Human Immuno-Deficiency Virus
ICF: Inner-City Fund
NPHRL: National Public Health Reference Laboratory
PLHIV: People Living With Human Immuno-Deficiency Virus
SDG: Sustainable Development Goal
SSA: Sub-Saharan Africa
STI: Sexually Transmitted Infection
VIF: Variance Inflation Factor
WHO: World Health Organization
