# Supplementary material for: Association between mass media exposure and endorsement of HIV-infected female teachers' teaching: insight from 2014 Ghana Demographic and Health Survey
Source: BMC Womens Health. 2022 Apr 15;22:121. doi: 10.1186/s12905-022-01705-1 (PMC9013040; doi:10.1186/s12905-022-01705-1)
Supplement: Supplementary file 1 — Additional file 1. Multi-collinearity test results. [file 12905_2022_1705_MOESM1_ESM.docx]

**Appendix S1: Multicollinearity test Result**

| Variable | VIF | 1/VIF |
| --- | --- | --- |
| Wealth status | 2.44 | 0.409835 |
| Residence | 1.80 | 0.554348 |
| Parity | 1.64 | 0.608174 |
| Education | 1.60 | 0.623606 |
| Age | 1.55 | 0.645856 |
| Access to Mass Media | 1.37 | 0.730678 |
| Partner’s education | 1.25 | 0.798371 |
| Religion | 1.15 | 0.868045 |
| Occupation | 1.06 | 0.944094 |
| Marriage | 1.06 | 0.946952 |
| Ethnicity | 1.02 | 0.980310 |
| Mean VIF | 1.45 |  |
